# Supplementary material for: Bre1/RNF20 promotes Rad51-mediated strand exchange and antagonizes the Srs2/FBH1 helicases
Source: Nat Commun. 2023 May 25;14:3024. doi: 10.1038/s41467-023-38617-z (PMC10213050; doi:10.1038/s41467-023-38617-z)
Supplement: Supplementary file 1 — Supplementary Information [file 41467_2023_38617_MOESM1_ESM.pdf]

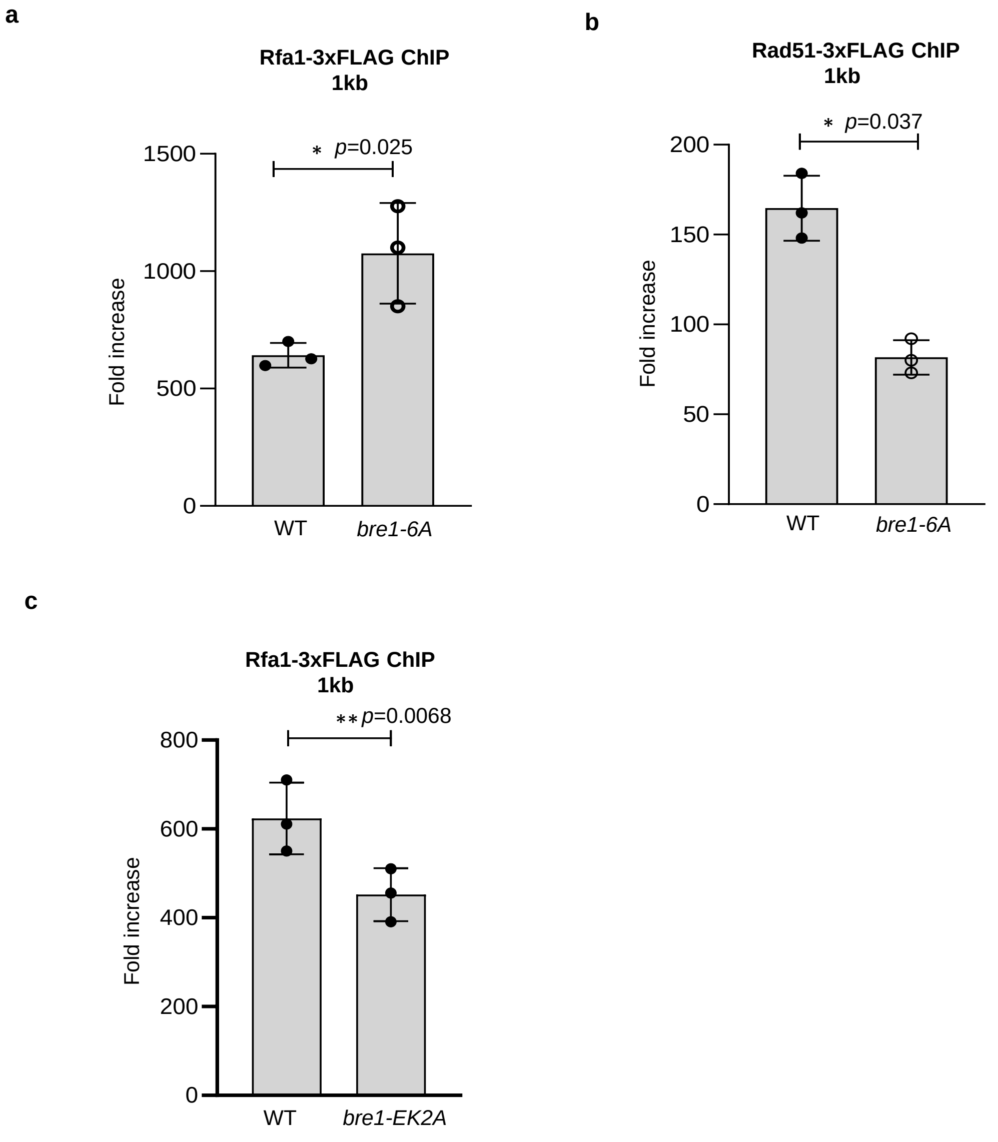

Supplementary Fig.1. Examination of the loading of RPA and Rad51 at DSBs. ChIP-qPCR showing the enrichment of Rfa1-3xFLAG (**a, c**) or Rad51-3xFLAG (**b**) at 1 kb away from the HO-induced DSB in indicated strains. Data in a-c are the mean ± SEM of three independent experiments (n=3). *$p<0.05$, **$p<0.01$ (Student *t*-test, two-tailed). Source data are provided as a Source Data file.

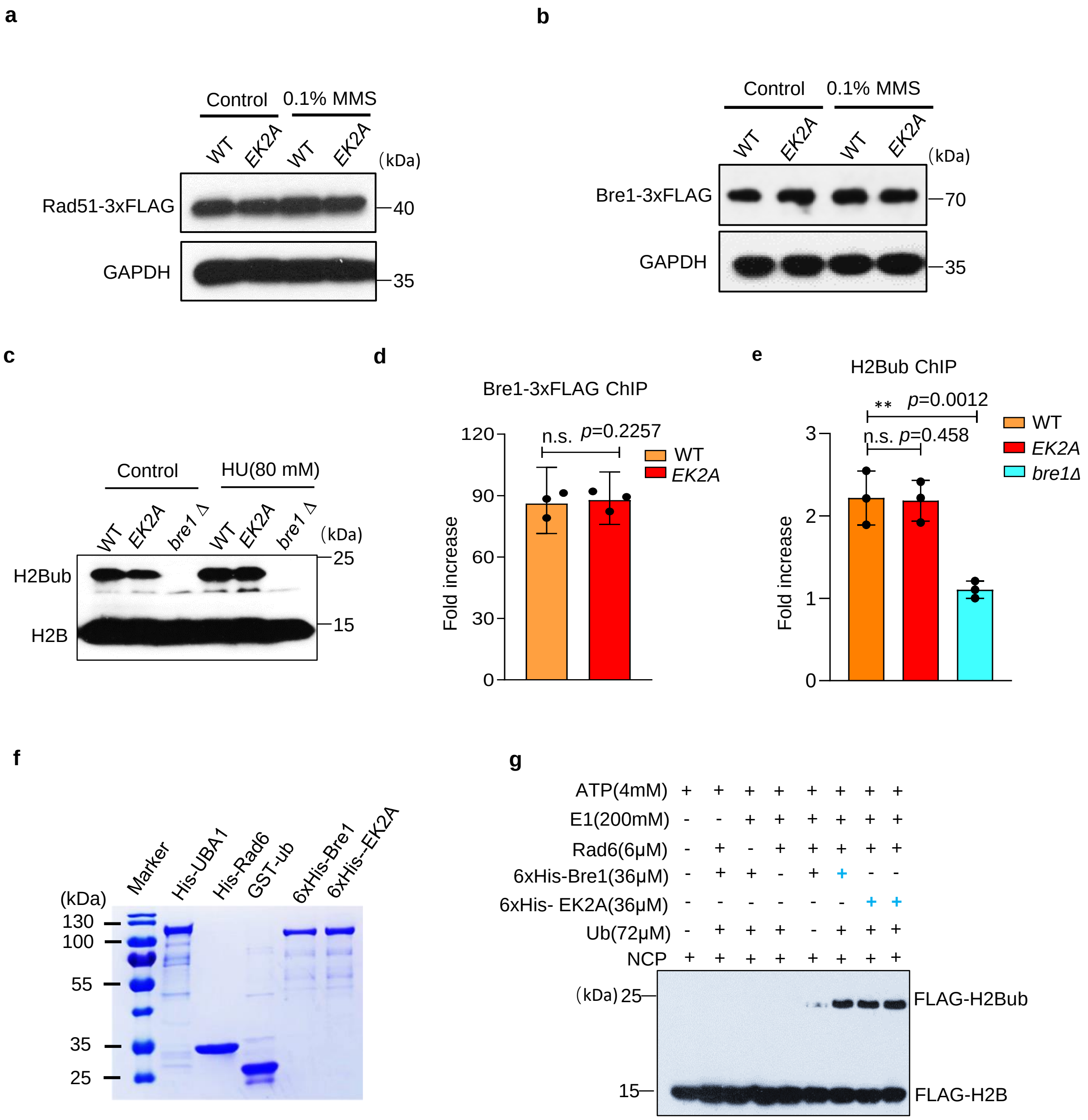

Supplementary Fig.2. The EK2A mutation does not affect the recruitment or ligase activity of Bre1. **(a-c)** Immunoblot showing the protein levels of Rad51, Bre1 and H2Bub, respectively, in indicated yeast cells. **(d-e)** ChIP-qPCR showing the enrichment of Bre1-3xFLAG (d) or H2Bub (e) at 1 kb away from the HO-induced DSB in indicated strains. Data are the mean ± SEM of three independent experiments (n=3). n.s, no significance; ***p*<0.01 (Student *t*-test, two-tailed). (**f**) Coomassie blue staining of purified proteins used for in vitro ubiquitination assay. (**g**) An in vitro ubiquitination assay followed by immunoblotting analysis showing the levels of H2B and H2Bub. Protein concentrations are indicated. Source data for panels (**d)** and (**e)** are provided as a Source Data file.

a

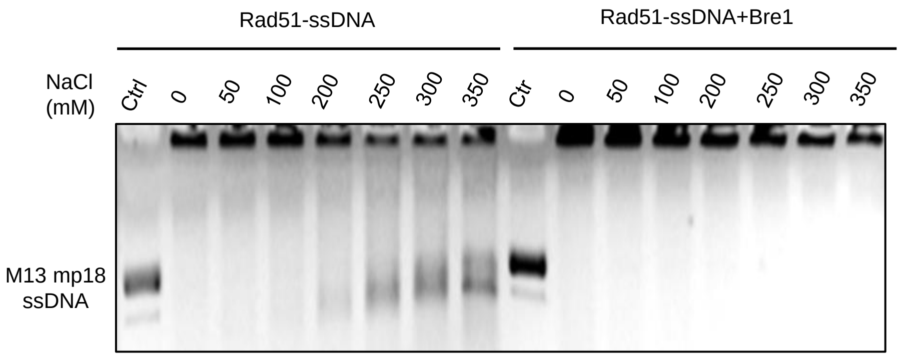

b

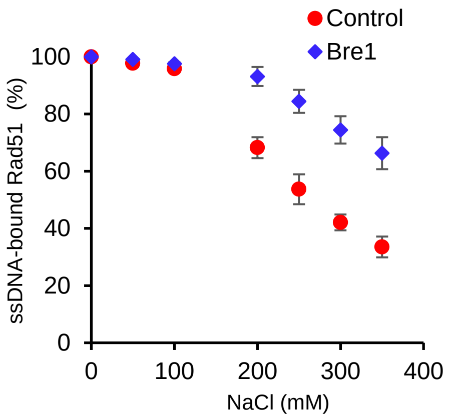

Supplementary Fig.3. Bre1 stabilizes the Rad51-ssDNA complexes under high salt. (a) Assessment of the effect of Bre1 on the stability of the Rad51-ssDNA complexes under different concentrations of NaCl. (**b**) Quantification of the ssDNA-bound Rad51 in (a). Data are the mean ± SD of three independent experiments (n=3). Source data for panel **(b)** are provided as a Source Data file.

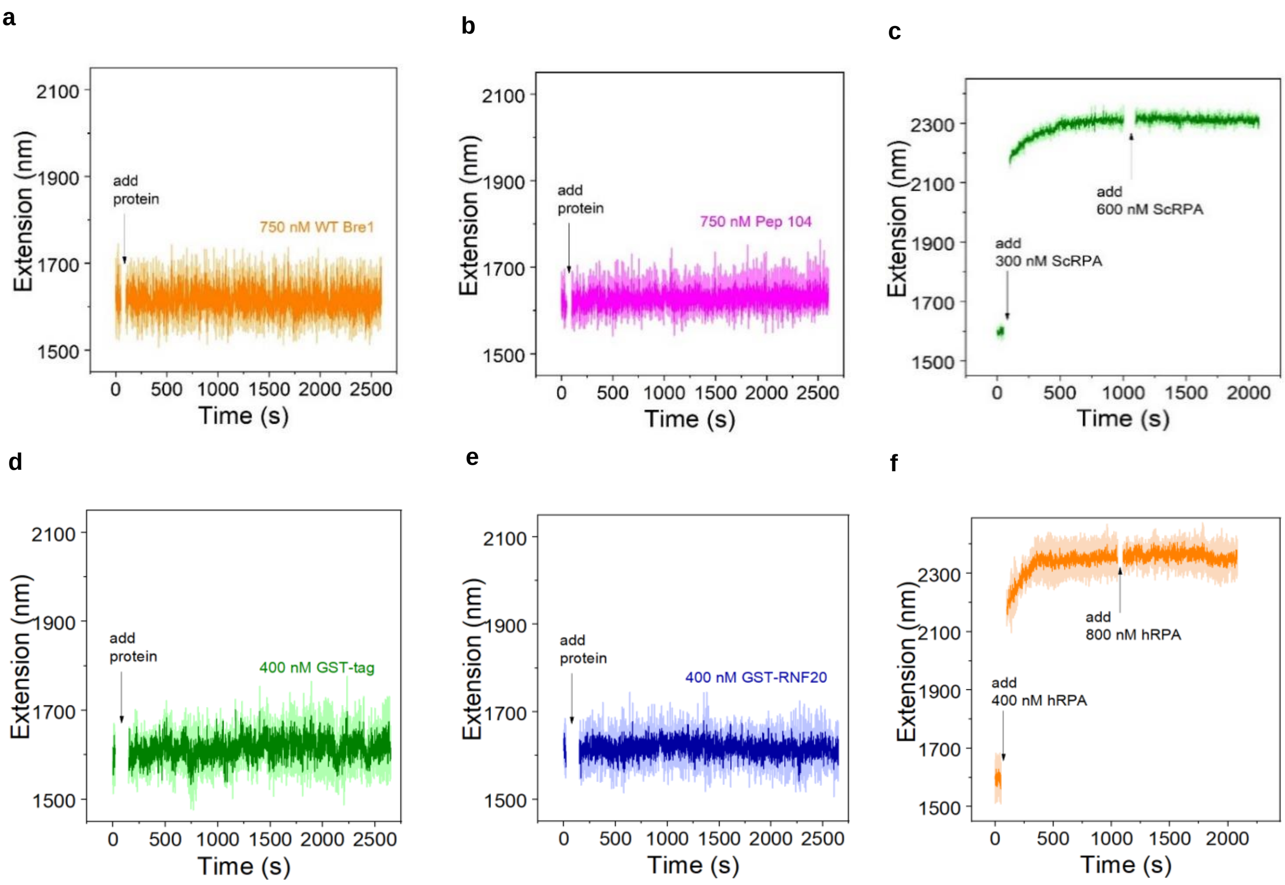

Supplementary Fig.4. Monitoring the effect of Bre1 and RNF20 on the extension of ssDNA by single-molecule MT. (a-b) The addition of Bre1 or Pep104 does not change ssDNA length. **(c)** Assessment of the saturation of ssDNA by the yeast RPA via single-molecule MT. The ssDNA was first incubated with 300 nM ScRPA for ~ 15 mins followed by the addition of 600 nM of ScRPA. (**d-e**) The addition of GST or GST-RNF20N does not alter ssDNA length. (**f**) Assessment of the saturation of ssDNA by the hRPA via single-molecule MT. The ssDNA was first incubated with 400 nM hRPA for ~ 20 mins followed by the addition of 800 nM of human RPA. The concentrations of proteins used are indicated. The reactions were carried out in the buffer with 10 mM MgAc, 100 mM NaAc, 25 mM Tris-Ac, pH 7.5, 0.02% BSA under 8 pN force at 21 ^{o}C. The average (dark-colored) and SD (lighter-colored) of the time courses at each condition in (**a-f)** were obtained from three independent molecules(n=3).

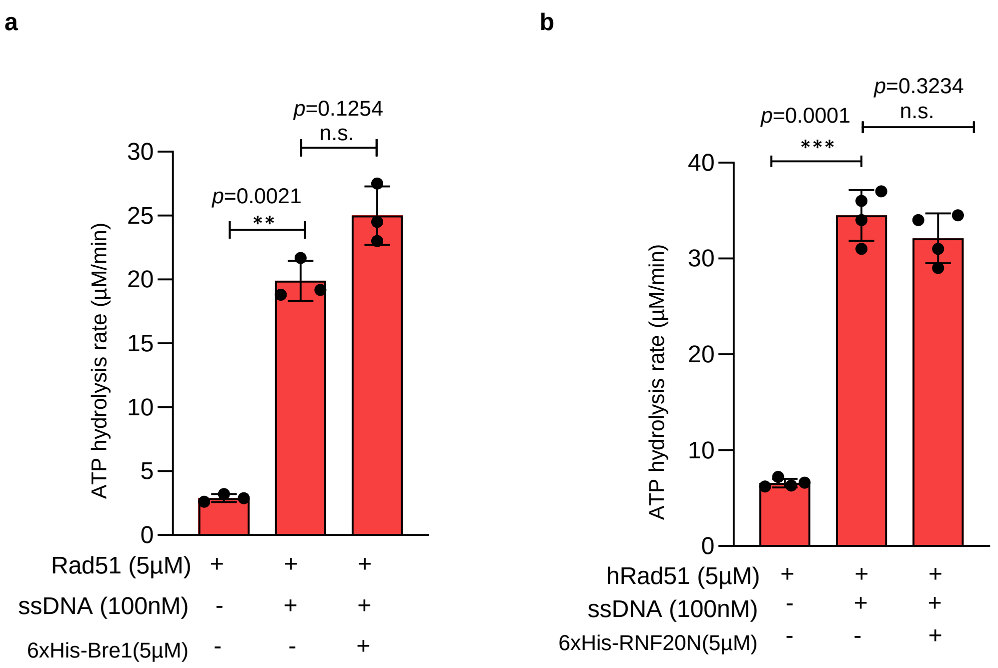

Supplementary Fig.5. The effect of Bre1 or RNF20 on the ATPase activity of the yeast Rad51(**a**) **or human hRad51(b**). Protein and ssDNA (M13 mp18) concentrations are indicated. Data are the mean ± SD of three independent experiments (n=3). n.s., no significance; ***p*<0.01, ****p*<0.001 (Student *t*-test, two-tailed). Source data are provided as a Source Data file.

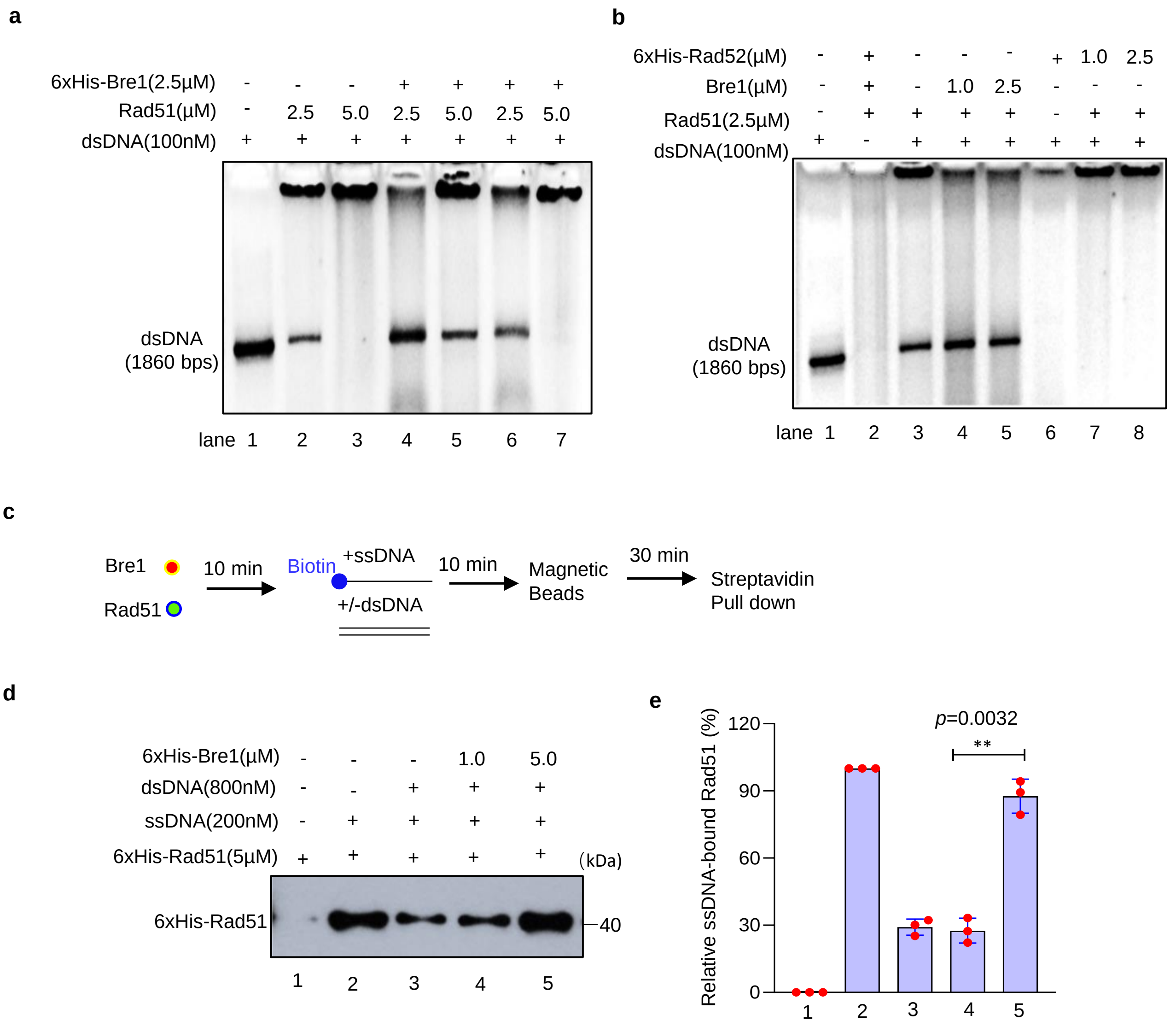

Supplementary Fig.6. Bre1 promotes Rad51 binding to ssDNA while restraining its loading onto dsDNA. (a-b) EMSA showing the effect of Bre1(a) or Rad52(b) on the binding of Rad51 to dsDNA. **(c)** Scheme showing an ssDNA pull-down assay. (**d**) An ssDNA pull-down assay showing the stimulation of Bre1 on Rad51 binding to ssDNA in the presence of competitive dsDNA (3788 bps). M13 mp18 ssDNA was used. Protein and DNA concentrations are indicated. (**e**) Quantification of the relative amount of the ssDNA-bound Rad51 showed in (d). The concentrations of proteins are indicated. Data are the mean ± SD of three independent experiments (n=3). $^{**}p<0.01$ (Student *t*-test, two-tailed). Source data for panel (**e**) are provided as a Source Data file.

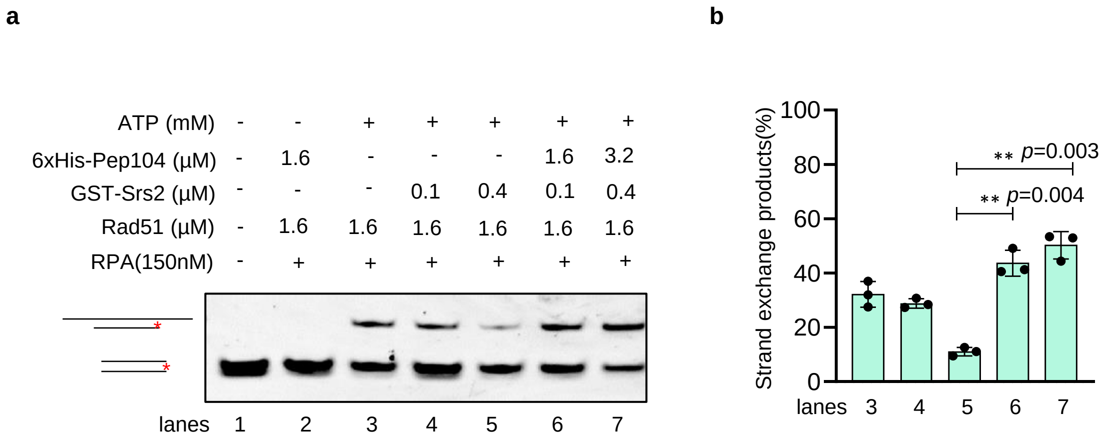

Supplementary Fig.7. The Bre1 peptide Pep104 is sufficient to repress the disrupting effect of Srs2 on strand exchange. a, An EMSA assay showing the effect of Bre1 on Rad51-mediated strand exchange. Protein concentrations are indicated. **b**, Quantification of the relative strand exchange products showed in **a**. Data are the mean ± SD of three independent experiments (n=3). $^{**}p<0.01$ (Student *t*-test, two-tailed). Source data for panel (**b)** are provided as a Source Data file.

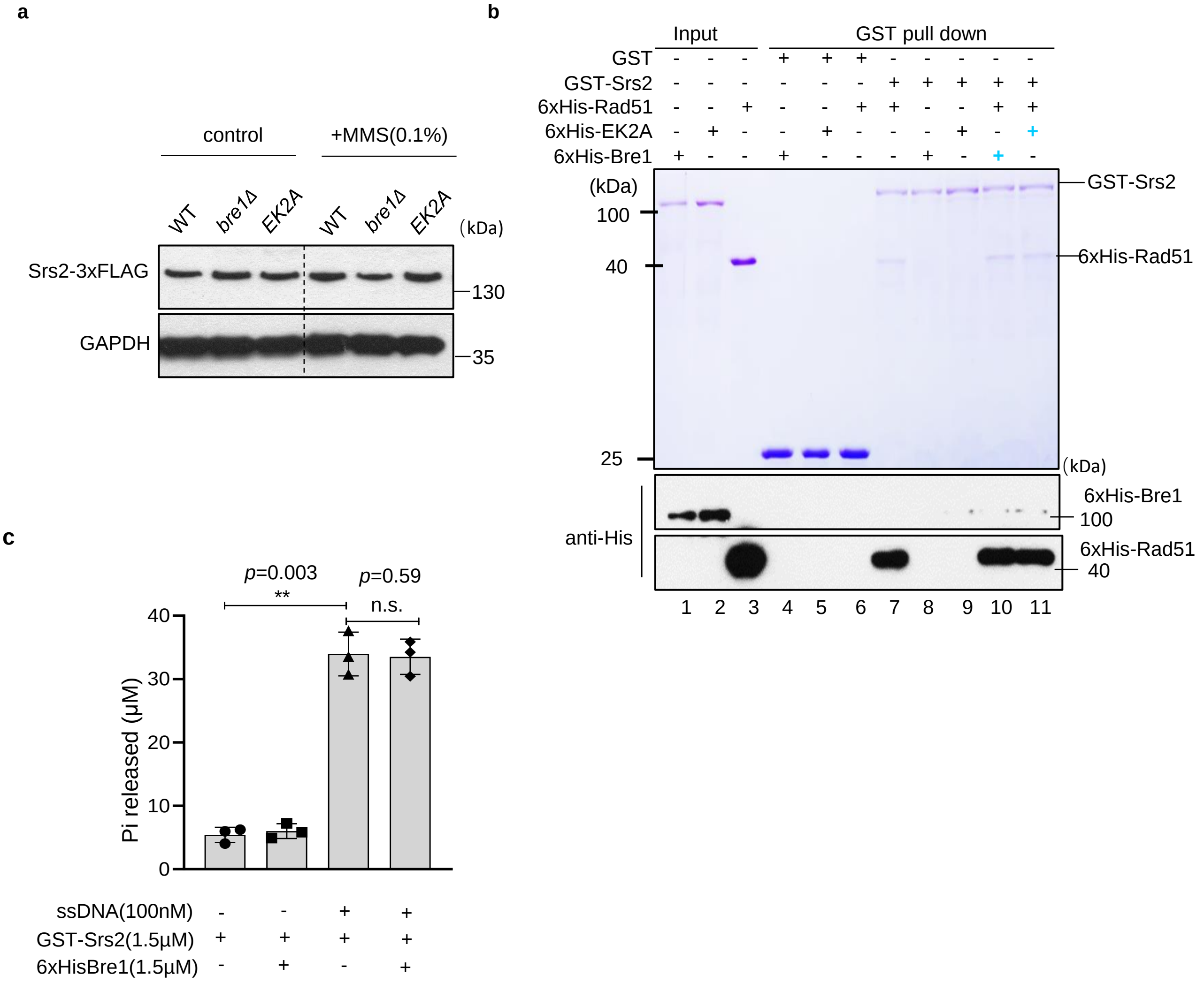

Supplementary Fig.8. Bre1 does not affect the Srs2-Rad51 interaction or the ATPase activity of Srs2. (a) Western blot analysis of Srs2-3xFLAG protein levels in indicated cells. **(b)** GST pull-down showing the interaction between GST-Srs2 and 6xHis-Rad51 in the presence or absence of 6xHis-Bre1 or 6xHis-bre1-EK2A protein (lane 7, 10 and 11). (**c**) The effect of Bre1 on the ATPase activity of Srs2. Data are the mean ± SD of three independent experiments (n=3). n.s., no significance; ***p*<0.01 (Student *t*-test, two-tailed). Source data for panel (**c**) are provided as a Source Data file.

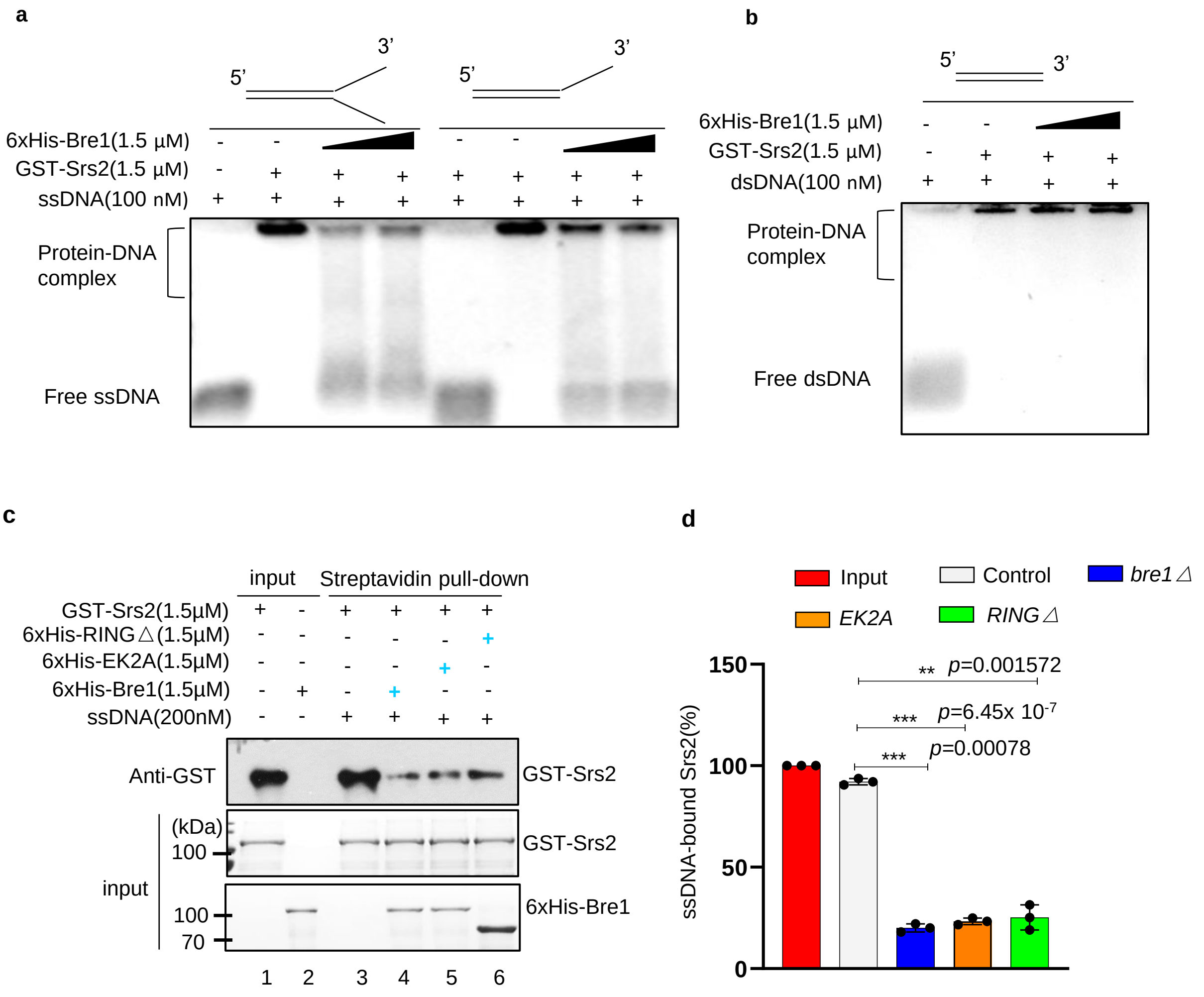

Supplementary Fig.9. Bre1 displaces Srs2 from ssDNA in an E3 ligase-independent manner. (a-b) Assessment of the effect of Bre1 on the binding of Srs2 to indicated DNA substrates by EMSA. Protein concentrations are indicated. Models for the DNA substrates are shown. **(c)** Assessment of the effect of the WT or mutant Bre1 protein on the binding of Srs2 to ssDNA substrates by an ssDNA pull-down assay. The products were detected by Western blot analysis with the anti-GST antibody. **(d)** Quantification of the relative amount of the ssDNA-bound Srs2 showed in (c). The concentration of DNA or protein used is indicated. Data are the mean ± SD of three independent experiments (n=3).). **p*<0.05, ***p*<0.01, ****p*<0.001 (Student *t*-test, two-tailed). Source data for panel (**d**) are provided as a Source Data file.

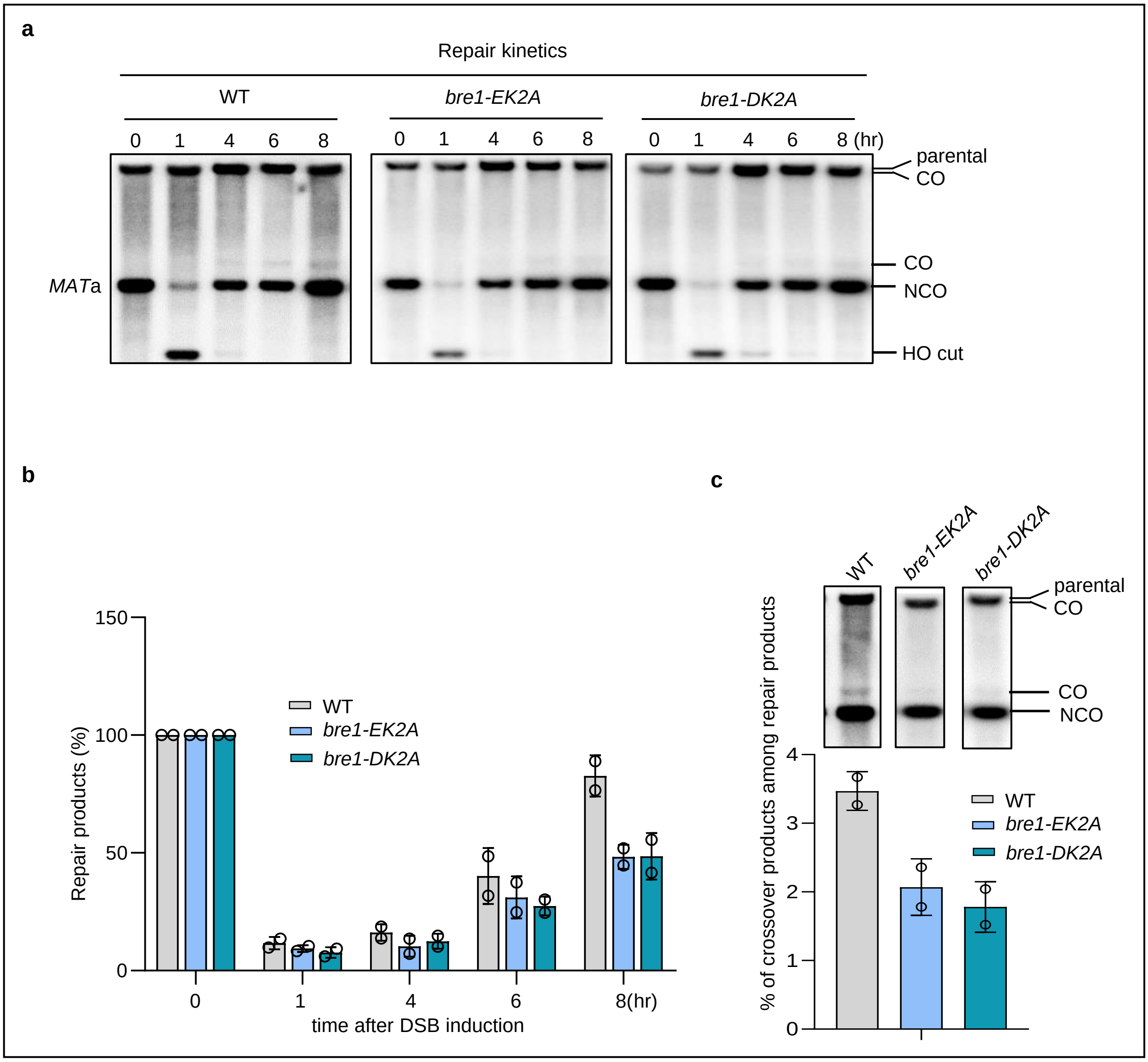

Supplementary Fig.10. The interaction between Bre1 and Rad51 or Srs2 is required for efficient HR repair. (a-b) Southern blot analysis and quantification of the ectopic recombination repair kinetics in the WT, *EK2A*, or *DK2A* cells. CO, crossover product; NCO, non-crossover product. (**c**) Plot showing the relative crossover level for indicated strains. Data in (**b**) and (**c**) are the mean ± SD of two independent experiments (n=2). Source data for panels (**b**) and **(c)** are provided as a Source Data file.

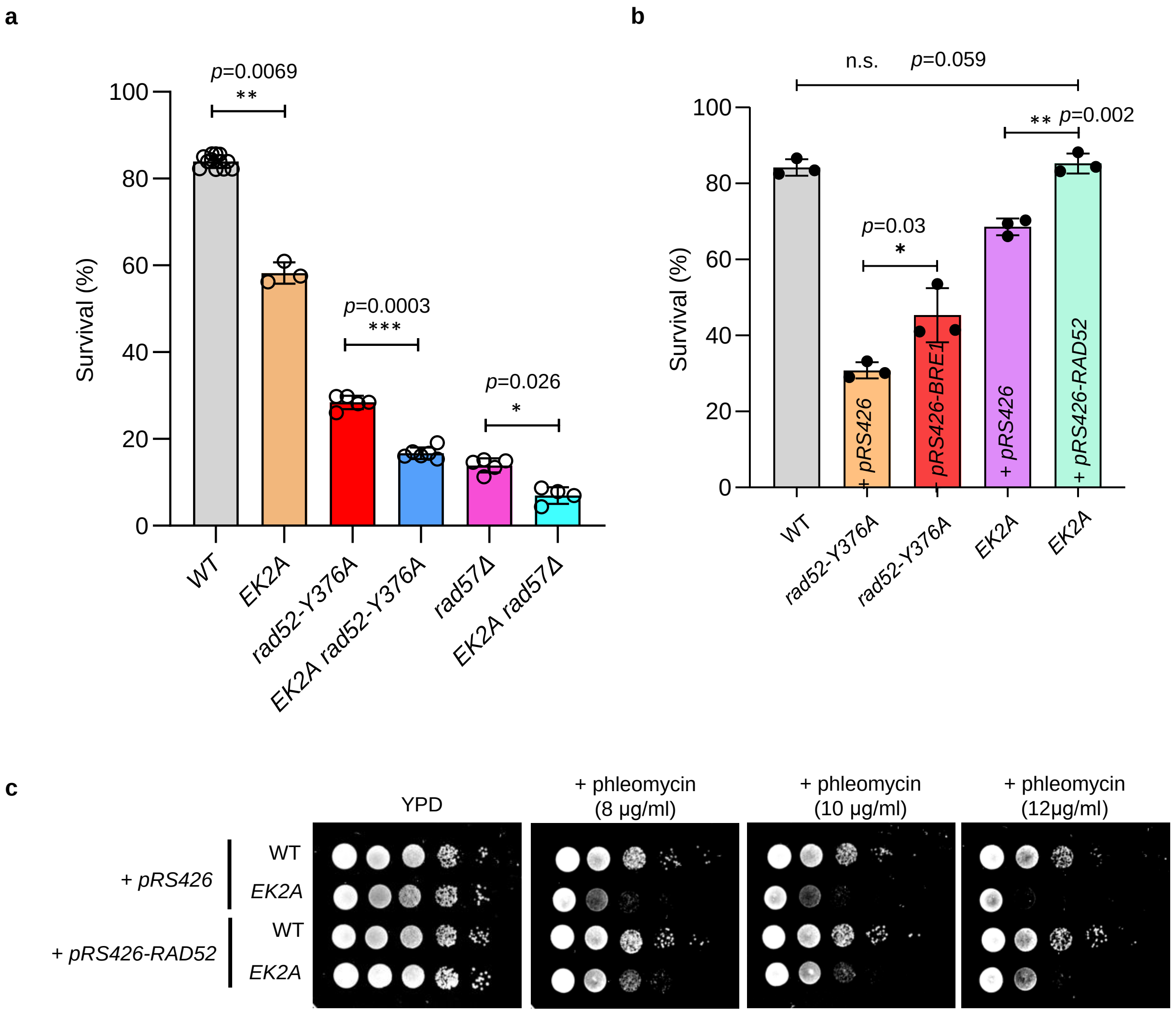

Supplementary Fig.11. Bre1 is additive to Rad52 or Rad55-Rad57 in DSB repair by ectopic recombination. (a-b) The survival rate of DSB repair by ectopic recombination in indicated strains. Data in a-b are the mean ± SEM of at least three independent experiments (n>3).). n.s., no significance; **p*<0.05, ** *p*<0.01, ****p*<0.001 (Student *t*-test, two-tailed). (**c**) DNA damage sensitivity test for indicated strains. Source data for panels (**a)** and (**b)** are provided as a Source Data file.

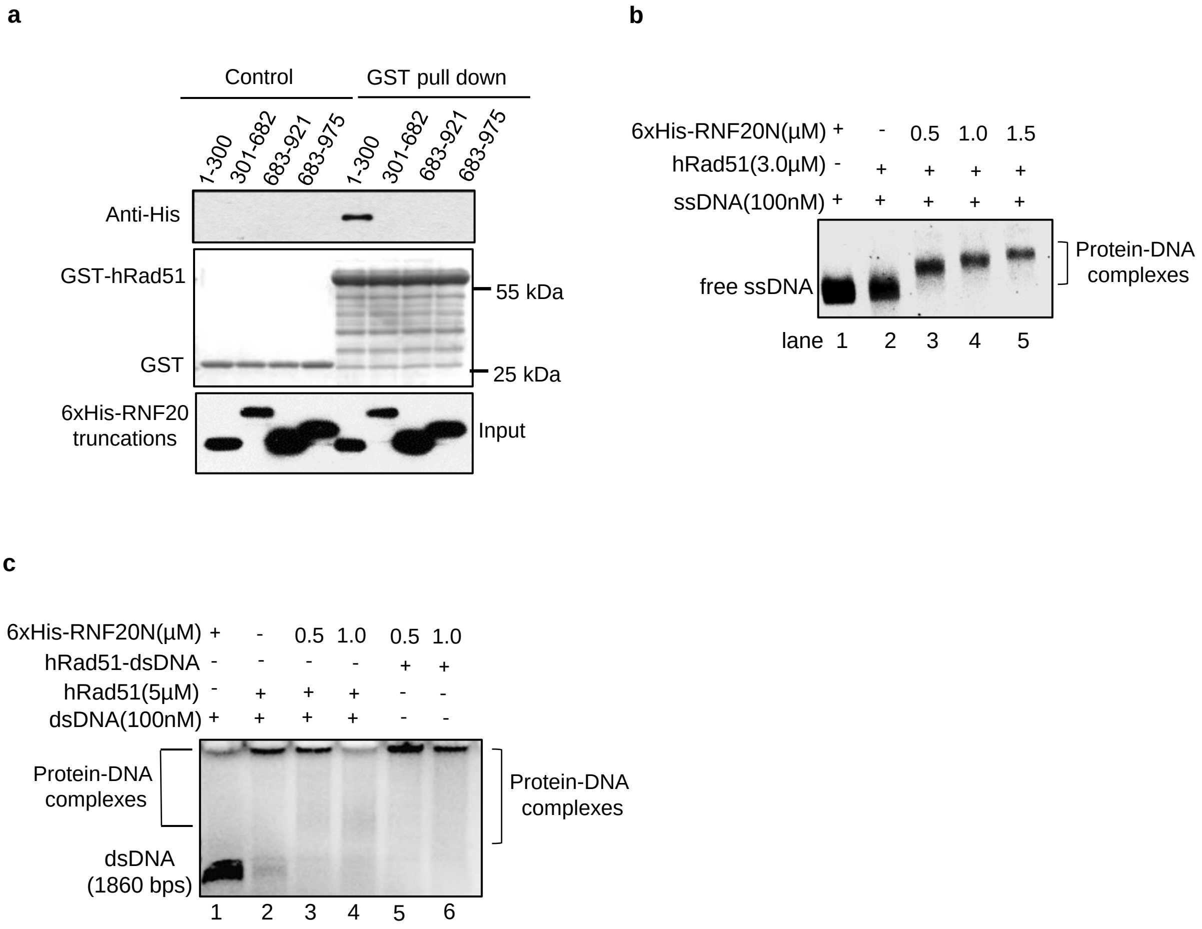

Supplementary Fig.12. RNF20 interacts with hRad51 and co-complexes with hRad51-ssDNA. (a) GST pull-down showing the interaction between GST-hRad51 and 6xHis-tagged truncated RNF20 proteins. **(b)** Assessment of the effect of RNF20N on hRad51 binding to ssDNA (M13 mp18) by EMSA. (**c**) Assessment of the effect of RNF20N on hRad51 binding to dsDNA by EMSA. The concentration of DNA or protein is indicated.

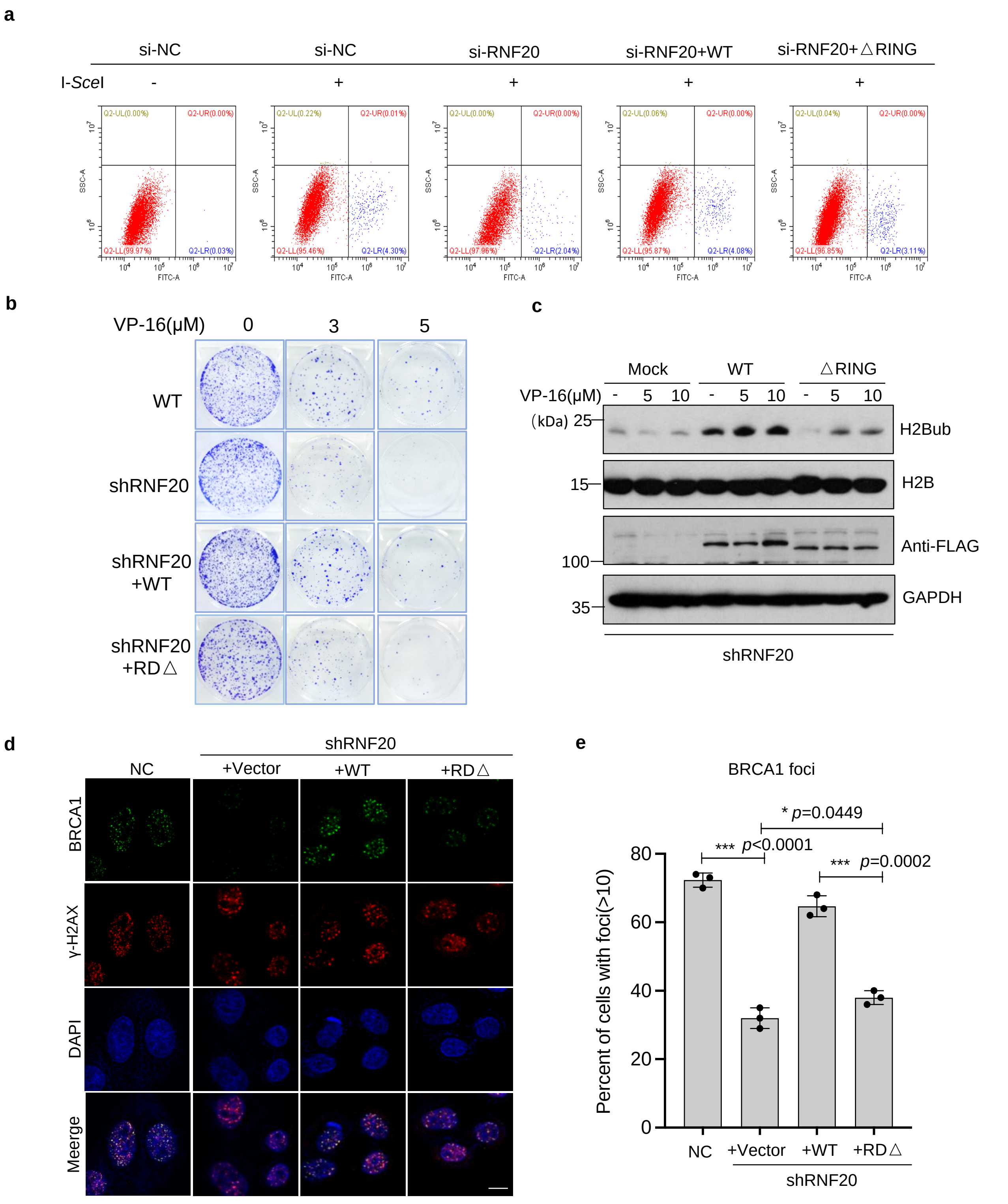

Supplementary Fig.13. RNF20 can promote HR repair and cell survival in an E3 ligase-independent manner upon DNA damage. (a) Comparison of HR repair efficiency in DR-GFP U2OS cells transfected with indicated siRNAs or a combination of siRNA and siRNA-resistant WT *RNF20* or *△RD* allele. Cells were harvested and subjected to flow cytometry analysis 48 hrs after transfection with I-*Sce*I. Representative images of GFP expression were shown. **(b)** Colony formation for indicated Hela cells following treatment with VP16 for 8hrs. **(c)** Western blot showing H2Bub level in indicated Hela cells. H2B and GAPDH were used as controls. **(d-e)** Immunostaining and quantification of BRCA1 foci formation at 1hr after VP-16 treatment in indicated Hela cells. Scale bar, 10 μm. Data are the mean ± SD of three independent experiments (n=3).). *p<0.05, ***p<0.001 (Student *t*-test, two-tailed). Source data for panel (**e**) are provided as a Source Data file.

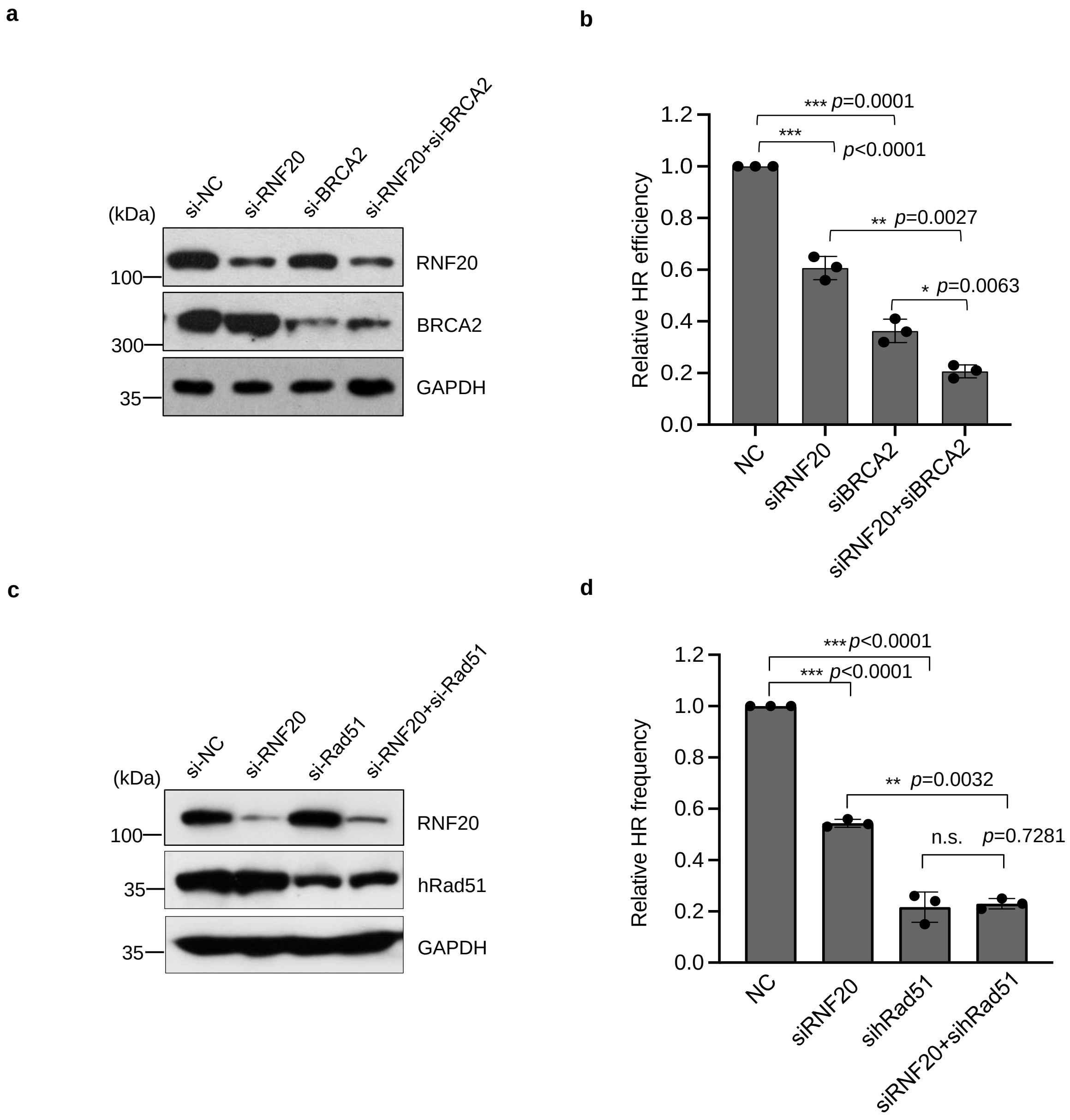

Supplementary Fig.14. RNF20 exhibits additive effect with BRCA2 on HR repair. (a, c) Western blot showing the levels of RNF20, BRCA2, or RAD51 in indicated U2OS cells. GAPDH serves as a loading control. **(b, d)** Plot showing the relative HR repair efficiency in indicated U2OS cells. Data in b and d are the mean ± SD of three independent experiments (n=3).). n.s., no significance; **p*<0.05, ** *p*<0.01, ****p*<0.001 (Student *t*-test, two-tailed). Source data for panels (**b)** and (**d)** are provided as a Source Data file.

a

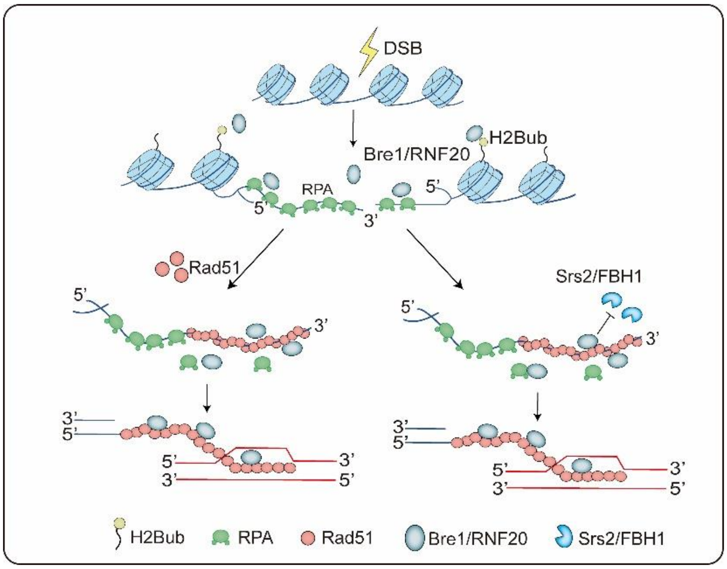

b

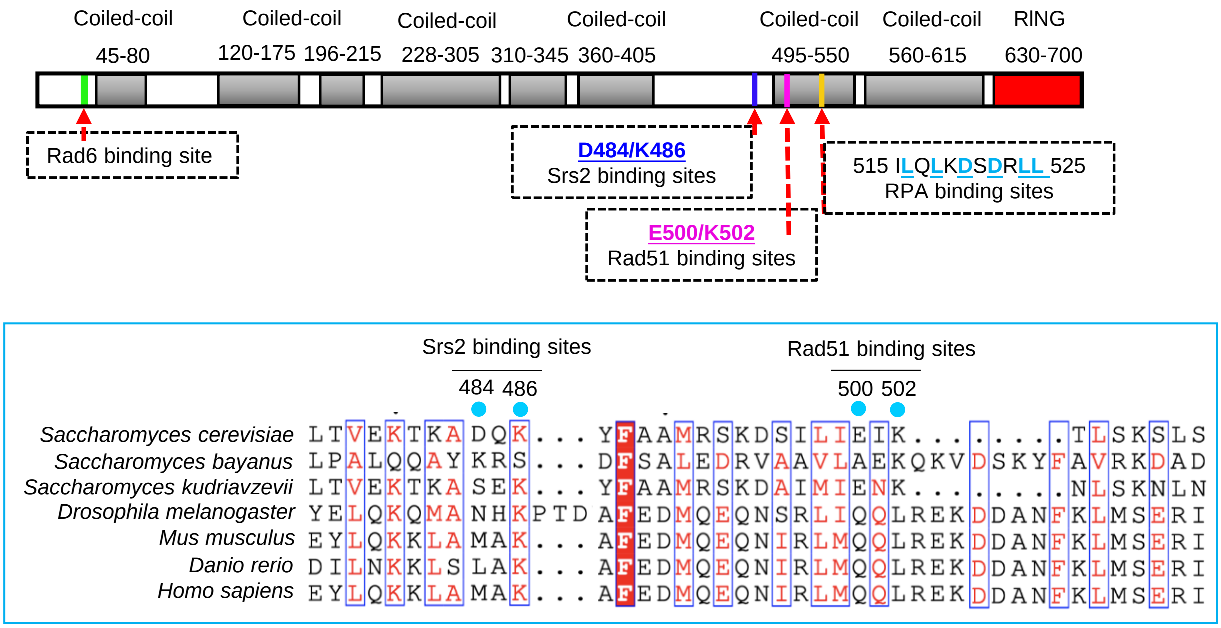

c

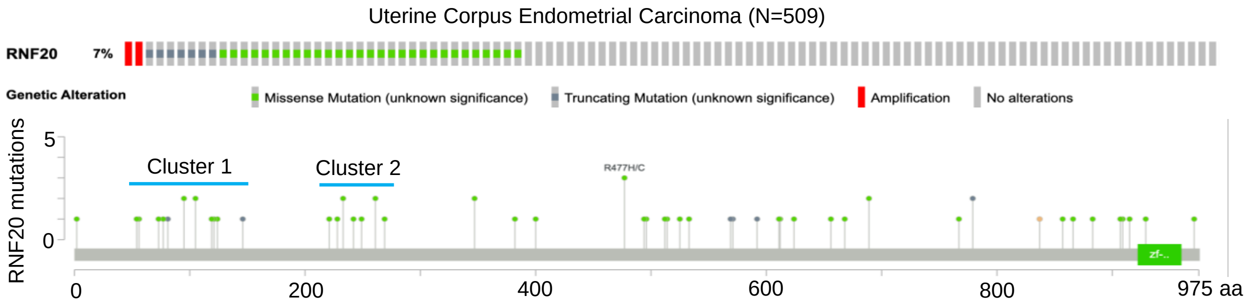

Supplementary Fig.15. **(a)** A work model illustrating the dual roles of Bre1/RNF20 in promoting HR. **(b)** Scheme showing the coiled-coil structures and the binding sites for Rad6, Srs2, Rad51 and RPA in Bre1 protein. The coiled coils were predicted with the online software COILS (http://www.expasy.org/resources/coils). The key residues mediating Bre1 interaction with Srs2, Rad51 or RPA are indicated. The Bre1 motif for mediating the interaction with Srs2 or Rad51 was aligned with Bre1 homologs from different species. The blue dots represent the key residues. (**c**) Scheme showing RNF20 mutations in uterine corpus endometrial carcinoma. The clustered mutations at the N-terminal end of RNF20 are marked.
