# Supplementary material for: Bre1/RNF20 promotes Rad51-mediated strand exchange and antagonizes the Srs2/FBH1 helicases
Source: Nat Commun. 2023 May 25;14:3024. doi: 10.1038/s41467-023-38617-z (PMC10213050; doi:10.1038/s41467-023-38617-z)
Supplement: Supplementary file 3 — Description of Additional Supplementary Files [file 41467_2023_38617_MOESM3_ESM.pdf]

Description of Additional Supplementary Files

File Name: Supplementary Data 1
Description: **List of primers, siRNA or shRNA sequences, yeast strains.**
Sheet 1: A list of primers used for plasmid construction or ChIP-qPCR.
Sheet 2: A list of sequences or resources for the siRNA or shRNA used in this study.
Sheet 3: Yeast strains used in this study.

File Name: Supplementary Data 2
Description: **A list of proteins identified in the proteomic study.**
