# Supplementary material for: Bre1/RNF20 promotes Rad51-mediated strand exchange and antagonizes the Srs2/FBH1 helicases
Source: Nat Commun. 2023 May 25;14:3024. doi: 10.1038/s41467-023-38617-z (PMC10213050; doi:10.1038/s41467-023-38617-z)
Supplement: Supplementary file 6 — Reporting Summary [file 41467_2023_38617_MOESM6_ESM.pdf]

nature portfolio

Corresponding author(s): Xuefeng Chen

Last updated by author(s): 26/3/23

Reporting Summary

Nature Portfolio wishes to improve the reproducibility of the work that we publish. This form provides structure for consistency and transparency in reporting. For further information on Nature Portfolio policies, see our Editorial Policies and the Editorial Policy Checklist.

Statistics

For all statistical analyses, confirm that the following items are present in the figure legend, table legend, main text, or Methods section.

n/a	Confirmed	
☐	☒	The exact sample size (*n*) for each experimental group/condition, given as a discrete number and unit of measurement
☐	☒	A statement on whether measurements were taken from distinct samples or whether the same sample was measured repeatedly
☐	☒	The statistical test(s) used AND whether they are one- or two-sided<br>*Only common tests should be described solely by name; describe more complex techniques in the Methods section.*
☒	☐	A description of all covariates tested
☒	☐	A description of any assumptions or corrections, such as tests of normality and adjustment for multiple comparisons
☐	☒	A full description of the statistical parameters including central tendency (e.g. means) or other basic estimates (e.g. regression coefficient) AND variation (e.g. standard deviation) or associated estimates of uncertainty (e.g. confidence intervals)
☐	☒	For null hypothesis testing, the test statistic (e.g. *F*, *t*, *r*) with confidence intervals, effect sizes, degrees of freedom and *P* value noted<br>*Give P values as exact values whenever suitable.*
☒	☐	For Bayesian analysis, information on the choice of priors and Markov chain Monte Carlo settings
☒	☐	For hierarchical and complex designs, identification of the appropriate level for tests and full reporting of outcomes
☒	☐	Estimates of effect sizes (e.g. Cohen's *d*, Pearson's *r*), indicating how they were calculated

Our web collection on statistics for biologists contains articles on many of the points above.

Software and code

Policy information about availability of computer code

Data collection	Data are presented as the means ± standard deviations (SDs).The coiled coils were predicted with the online software COILS (http://www.expasy.org/resources/coils). Software was used for collection and analysis and is listed below.
Data analysis	The StepOne™ Software for qPCR; The Leica Application Suite X and ZEISS Blue Lite2 software for Immunofluorescence or live cells examined; The CytExpert for flow cytometry;The GraphPad Software(version 8.0) and ImageJ (version 1.8.0) for quantitative and statistical analysis; The CytExpert for flow cytometry.

For manuscripts utilizing custom algorithms or software that are central to the research but not yet described in published literature, software must be made available to editors and reviewers. We strongly encourage code deposition in a community repository (e.g. GitHub). See the Nature Portfolio guidelines for submitting code & software for further information.

Data

Policy information about availability of data

All manuscripts must include a data availability statement. This statement should provide the following information, where applicable:

- Accession codes, unique identifiers, or web links for publicly available datasets
- A description of any restrictions on data availability
- For clinical datasets or third party data, please ensure that the statement adheres to our policy

All data associated with this study are presented in the paper or the supplementary information.

Human research participants

Policy information about studies involving human research participants and Sex and Gender in Research.

Reporting on sex and gender	N/A
Population characteristics	N/A
Recruitment	N/A
Ethics oversight	N/A

Note that full information on the approval of the study protocol must also be provided in the manuscript.

Field-specific reporting

Please select the one below that is the best fit for your research. If you are not sure, read the appropriate sections before making your selection.

☒ Life sciences ☐ Behavioural & social sciences ☐ Ecological, evolutionary & environmental sciences

For a reference copy of the document with all sections, see nature.com/documents/nr-reporting-summary-flat.pdf

Life sciences study design

All studies must disclose on these points even when the disclosure is negative.

Sample size	No statistical method was used to predetermine sample size, but we routinely employed at least three biological repeats for each experiment, in each case scoring as many technical replicates as possible (typically several hundred) .
Data exclusions	no data was excluded
Replication	all findings were independently replicated at least 3 times. All replicates were successful and included in the data
Randomization	Samples were not randomized, as it was either not applicable or not necessary due to automated and unbiased analysis. Samples were separated in groups based on different genetic backgrounds or whether or not they were treated or not treated (small molecule inhibitors; siRNA, etc.)
Blinding	For manual microscopy, data were collected randomly choose at least 3 region. The investigators who collected and analyzed data were blind to group allocation. For flow cytometry and microscopy, data were collected automatically by the equipments. The investigators who operated these equipments were blind to group allocation. One individual performed the sample treatment while another individual (blinded to the group allocation) performed the analysis. For other cell experiments , investigators collecting data or analyzing data were blind to group allocation or sample treatment.

Reporting for specific materials, systems and methods

We require information from authors about some types of materials, experimental systems and methods used in many studies. Here, indicate whether each material, system or method listed is relevant to your study. If you are not sure if a list item applies to your research, read the appropriate section before selecting a response.

Materials & experimental systems

n/a	Involved in the study
☐	☒ Antibodies
☐	☒ Eukaryotic cell lines
☒	☐ Palaeontology and archaeology
☒	☐ Animals and other organisms
☒	☐ Clinical data
☒	☐ Dual use research of concern

Methods

n/a	Involved in the study
☒	☐ ChIP-seq
☐	☒ Flow cytometry
☒	☐ MRI-based neuroimaging

Antibodies

Antibodies used

Western blot:
anti-FLAG (Cell Signaling Technology; Rabbit #14793; 1:1000 dilution)
anti-H2Bub(Cell Signaling Technology;Rabbit #5546;1:1000 dilution)
anti-RNF20(abcam; Rabbit; ab32629; 1:3000 dilution)
anti-FBH1(abcam; Mouse; ab58881; 1:1000 dilution)
anti-Rad51(abcam; Rabbit; ab133534; 1:3000 dilution)
anti-RPA32(abcam; Mouse; ab2175; 1:1000 dilution)
anti-anti-Brca2(ABclona;Rabbit;A2435; 1:1000 diluton)
anti-HA(ABclona;Mouse; AE008; 1:5000 dilution)
anti-H2B(ABclona;Rabbit; A1958;1:5000 dilution)
anti-GAPDH (ABclona;Mouse;AC002,1:5000 dilution)
anti-His(Proteintech;Mouse;66005-1-Ig; 1:5000 dilution)
anti-GST (Proteintech;Mouse;66001-2-Ig; 1:5000 dilution)
Horseradish peroxidase (HRP) conjugated secondary antibodies used were uesed:
HRP Goat anti-mouse IgG(Jackson immuno #115-035-003, 1:10000)
HRP Goat anti-Rabbit IgG(Jackson immuno #111-035-003, 1:10000)

Immunostaining
anti-gamma-H2AX (abcam;Mouse;ab22551;1:100 dilution)
anti-RPA2(abcam;Mouse;ab2175; 1:200 dilution)
anti-Rad51(abcam;Rabbit;ab133534; 1:400 dilution)
anti-Brca1(Santa Cruz Biotechnology;Mouse;sc-6954; 1:100 dilution)
anti-FLAG(Cell Signaling Technology;Rabbit;#14793; 1:200 dilution)
The following secondary antibodies were used:
Alexa Fluor 488 Goat Anti-Mouse (Life Technologies, A-11008, 1:1000)
Alexa Fluor 594 Goat Anti-Rabbit (Life Technologies, A-11012, 1:1000)

Validation

All antibodies are are commercially available and validated by manufacturers via western blot analysis and immunofluorescence as documented in the manufacturer's websites.

Eukaryotic cell lines

Policy information about cell lines and Sex and Gender in Research

Cell line source(s)

HEK293T (#CRL-3216) and HeLa (#CCL-2) cells were purchsed from ATCC. The U20S-DR reporter cell line generated from U2OS (kindly provided by Dr. Xingzhi Xu)(Shenzhen University) was originally purchased from ATCC.

Authentication

HEK293T and HeLa cells were purchased from the credible vendors, and they provided the authetication file about STR assay.The U20S-DR (gift from Dr. Xingzhi Xu) was not further authenticated.

Mycoplasma contamination

all cells were confirmed mycoplasma negative.

Commonly misidentified lines
(See ICLAC register)

no commonly misidentified cell lines were employed in this study

Flow Cytometry

Plots

Confirm that:

☒ The axis labels state the marker and fluorochrome used (e.g. CD4-FITC).

☒ The axis scales are clearly visible. Include numbers along axes only for bottom left plot of group (a 'group' is an analysis of identical markers).

☒ All plots are contour plots with outliers or pseudocolor plots.

☒ A numerical value for number of cells or percentage (with statistics) is provided.

Methodology

Sample preparation	DR-U20S cells were transfected spcific siRNA were analysis by FACS for GFP expression accumulated 48 hr after I-SceI expression.
Instrument	Data were collected on CytoFlex S (Beckman Coulter)
Software	CytExpert
Cell population abundance	At least 10000 gated cells were collected for each groups.
Gating strategy	Cells were first gated for FSC/SSC,then successfully repaired cells as indicated in the figure legends.

☒ Tick this box to confirm that a figure exemplifying the gating strategy is provided in the Supplementary Information.
